# Filling the Gaps in the LiBr-LiOH Phase Diagram: A Study on the High-Temperature Li_3_(OH)_2_Br Phase

**DOI:** 10.1021/acs.chemmater.5c00206

**Published:** 2025-04-04

**Authors:** Emily Milan, James A. Quirk, Kenjiro Hashi, John Cattermull, Andrew L. Goodwin, James A. Dawson, Mauro Pasta

**Affiliations:** † Department of Materials, University of Oxford, Oxford OX1 3PH, U.K.; ‡ Chemistry—School of Natural and Environmental Sciences, Newcastle University, Newcastle upon Tyne NE1 7RU, U.K.; § National Institute for Materials Science, Tsukuba 305-0044, Japan; ∥ Department of Chemistry, University of Oxford, Oxford OX1 3QR, U.K.

## Abstract

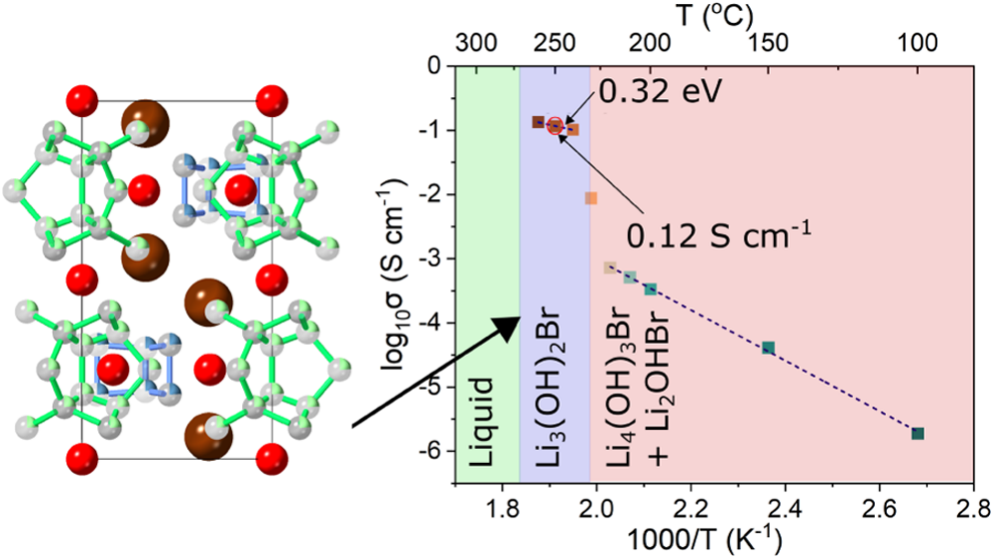

In this paper, we build on previous work to characterize a phase with stoichiometry Li_3_(OH)_2_Br existing between ∼225 and ∼275 °C in the LiBr-LiOH phase diagram. Diffraction studies indicate that the phase takes a hexagonal unit cell, and theoretical modeling is used to suggest a possible crystal structure. Nuclear magnetic resonance spectroscopy and electrochemical impedance spectroscopy measurements demonstrate excellent lithium-ion dynamics in this phase, with an ionic conductivity of 0.12 S cm^–1^ at 250 °C. Initial attempts to stabilize this phase at room temperature through quenching were not successful. Instead, a metastable state demonstrating poor ionic conductivity is found to form. This is an important consideration for the synthesis of Li_2_OHBr solid-state electrolytes (also found in the LiBr-LiOH phase diagram) which are synthesized by cooling through phase fields containing Li_3_(OH)_2_Br, and are hence susceptible to these impurities.

## Introduction

Recently, materials in the LiBr-LiOH system have received significant attention in energy storage research: Li_2_OHBr as a promising solid-state electrolyte for lithium–metal batteries, and Li_4_(OH)_3_Br as a phase change material for thermal energy storage.^1−10^ Initial work by Scarpa and Hartwig believed these to be the only compounds existing within the LiBr-LiOH phase diagram;^11,12^ however, a high-temperature phase with stoichiometry Li_3_(OH)_2_Br was mentioned by Reshetnikov in 1953.^13^ No information about the phase is available, and in 2000, Sangster chose to omit it from their proposed phase diagram.^14^

**Figure 1:**
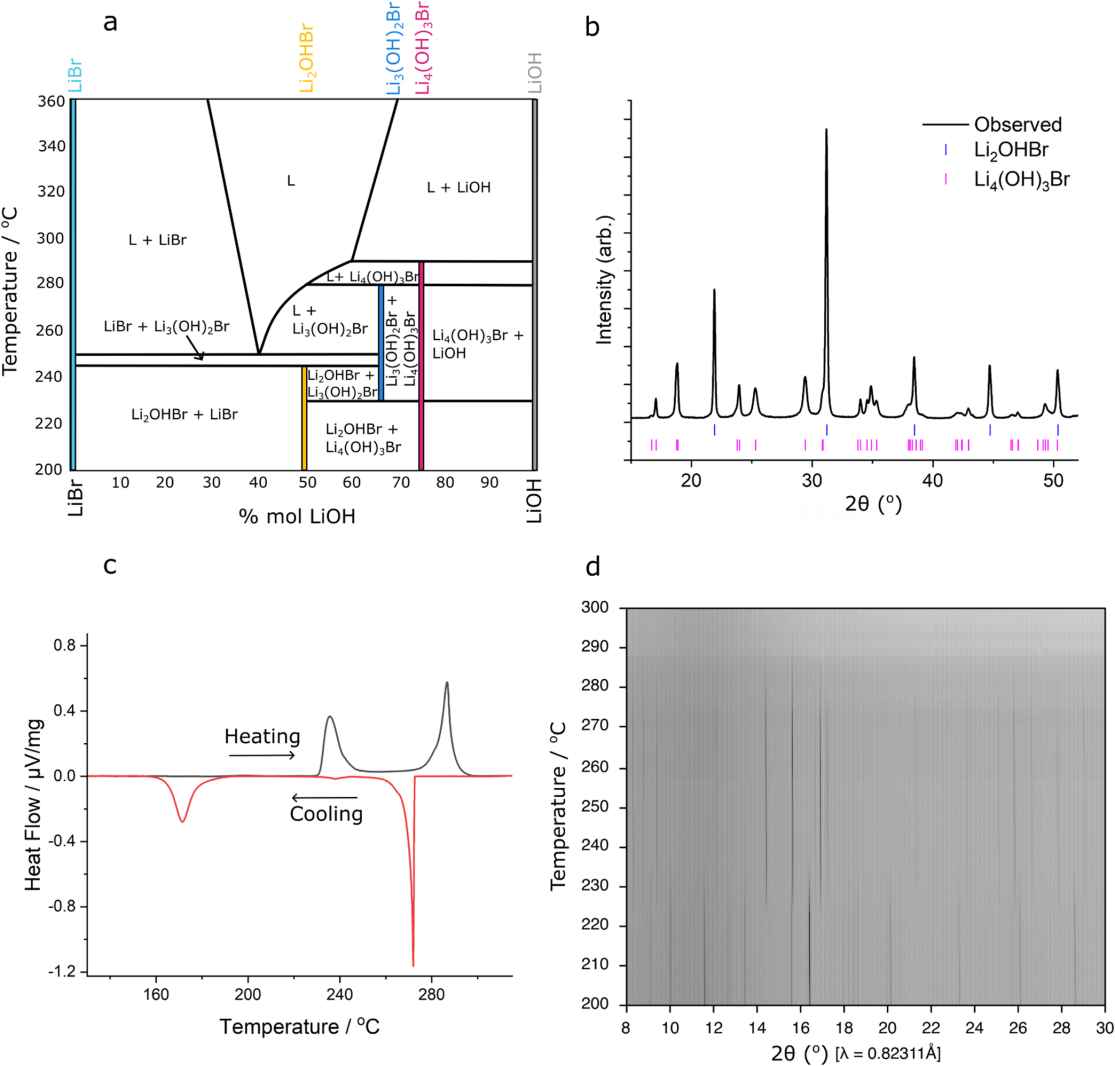
Existence of Li_3_(OH)_2_Br. (a) LiBr-LiOH phase diagram proposed by Mahroug et al.,^9^ drawn here with phase-field labels. Stoichiometric compounds LiBr, Li_2_OHBr, Li_3_(OH)_2_Br, Li_4_(OH)_3_Br, and LiOH are indicated with colored lines. Li_3_(OH)_2_Br is included as a peritectic compound at 67 mol % LiOH, between ∼230 and ∼280 °C. The widths of lines corresponding to stoichiometric compounds are not representative of their stoichiometric ranges, which is unknown. (b) XRD pattern from room-temperature 67 mol % LiOH sample cooled from 350 °C at 3 °C/min. Bragg peak positions for Li_4_(OH)_3_Br and Li_2_OHBr phases are indicated by ticks beneath the data. (c) Heating and cooling DSC of 67 mol % LiOH sample at a rate of 5 °C/min. Two peaks are seen in each instance, expected to correspond to the formation and decomposition of Li_3_(OH)_2_Br. (d) Film plot showing synchrotron variable-temperature XRD measurements (λ = 0.82311 Å) of the 67 mol % LiOH sample shown in part b, heated at 6 °C/min. A phase transition occurs at ∼225 °C, followed by melting from ∼270 °C.

In 2022, Mahroug et al. investigated to substantiate claims of a Li_3_(OH)_2_Br phase.^9^ Crucially, differential scanning calorimetry (DSC) measurements taken across a range of compositions indicated a phase change at ∼230 °C which had not previously been identified, with a maximum transition enthalpy for the composition 67 mol % LiOH, indicating that the stoichiometry of the unknown phase lies at this LiBr-LiOH ratio. They additionally conducted in situ and ex situ X-ray diffraction (XRD) measurements which showed the formation of new diffraction reflections upon heating samples from the room-temperature “Li_2_OHBr + Li_4_(OH)_3_Br” phase field, although the validity of these observations is unknown since their starting Li_4_(OH)_3_Br has since been shown to be a metastable hydrated phase by Milan et al.^15^ Nevertheless, these findings suggest that there may be a compound at this composition. Based on these findings, Mahroug et al. developed the existing phase diagram to include Li_3_(OH)_2_Br. As shown in Figure 1a, these alterations would introduce Li_3_(OH)_2_Br as a peritectic phase (liquid + Li_4_(OH)_3_Br → Li_3_(OH)_2_Br).^9^

Understanding the LiBr-LiOH phase diagram may be critical to understanding impurity formation in Li_2_OHBr and solidification phenomena in Li_4_(OH)_3_Br, as well as the possibility of exciting properties offered by the discovery of novel materials. In this paper, the existence of a Li_3_(OH)_2_Br phase is confirmed, and the phase is characterized for the first time. We use a combination of diffraction studies and modeling to determine a possible crystal structure. Its lithium-ion dynamics are evaluated through the use of nuclear magnetic resonance (NMR) spectroscopy and electrochemical impedance spectroscopy (EIS), and supported further by molecular dynamics (MD) simulations. We find that this phase cannot easily be retained at room temperature, instead forming a metastable state. The properties of this material are also considered, and its implications are addressed.

## Results and Discussion

### Existence of Li_3_(OH)_2_Br

Anhydrous LiBr-LiOH was ground together in the correct stoichiometric ratio, 66.7 mol % LiOH to 33.3 mol % LiBr, and heated at 5 °C/min to its molten state at 350 °C for 30 min. Upon furnace cooling at 3 °C/min to room temperature, a material containing Li_2_OHBr and the anhydrous Li_4_(OH)_3_Br phase reported by Milan et al. is formed (Figures 1b and S1).^15^ This material was ground into a powder using a mortar and pestle and investigated by DSC and variable-temperature (VT) XRD from room temperature to 300 °C. Figure 1c,d shows these DSC and VT-XRD measurements, taken at 5 and 6 °C/min, respectively. In both instances, a phase transition to a new crystalline phase is observed at ∼230 °C, as expected from Mahroug’s phase diagram.

This phase persists until melting. The first indications of melting onset between 260 and 270 °C, where a uniform reduction in XRD peak intensity and rapid narrowing of the ^7^Li NMR line shape occurs (Figure S2), with the Li_3_(OH)_2_Br phase fully disappearing by ∼290 °C. Unlike indicated in Mahroug’s phase diagram, no Li_4_(OH)_3_Br is observed prior to melting. Additionally, on further heating to 300 °C, no LiOH forms, as expected from the phase diagram, but instead another peak appears, as of yet unidentified. It is difficult to ascertain whether these observations correspond to equilibrium conditions and hence indicate a discrepancy with the phase diagram, or can be attributed to nonequilibrium phenomena. Nevertheless, our findings support the existence of a phase with composition Li_3_(OH)_2_Br which forms from 225 °C upon heating and melts around 270 °C.

### Structural Characterization

A cold-pressed pellet of the 67 mol % LiOH sample was heated to 250 °C at 1 °C/min. To ensure equilibrium conditions, the sample was held at 250 °C for 1 h under in situ XRD. The diffraction pattern was found to be stable in this time, indicating that equilibrium conditions were being obtained and what is believed to be a single phase. The observed peak positions for the Li_3_(OH)_2_Br phase were indexed and space group searching was conducted using TOPAS-Academic software^16^ (Table S1). The peaks were found to be fit well by several space groups with hexagonal unit cells. A Pawley refinement for the *P*6_3_ space group, shown in Figure S3 (Table S2), fits the diffraction pattern in excellent agreement, giving lattice parameters of *a* = *b* = 6.57192(6) Å, *c* = 10.74643(17) Å.

A low-energy structure for Li_3_(OH)_2_Br was determined computationally through ab initio random structure searching. Thousands of structures were generated in which the Li–O–Br sublattice was arranged in the *P*6_3_ space group, with H ignored in symmetry determination as it could not be refined in XRD. Each candidate structure was optimized using a CHGNet machine-learned force field fine-tuned for the Li–O–H–Br phase space.^17^ A possible structure for Li_3_(OH)_2_Br was found and equilibrated at 250 °C so that fractional occupations at a finite temperature could be determined. The lithium atoms were found to be highly mobile, meaning that precise occupancies could not be determined from MD. As such, the occupancies have been assumed to be equal across all sites. The resultant structure is shown in Figure S4, and it is detailed in Table S3.

**Figure 2:**
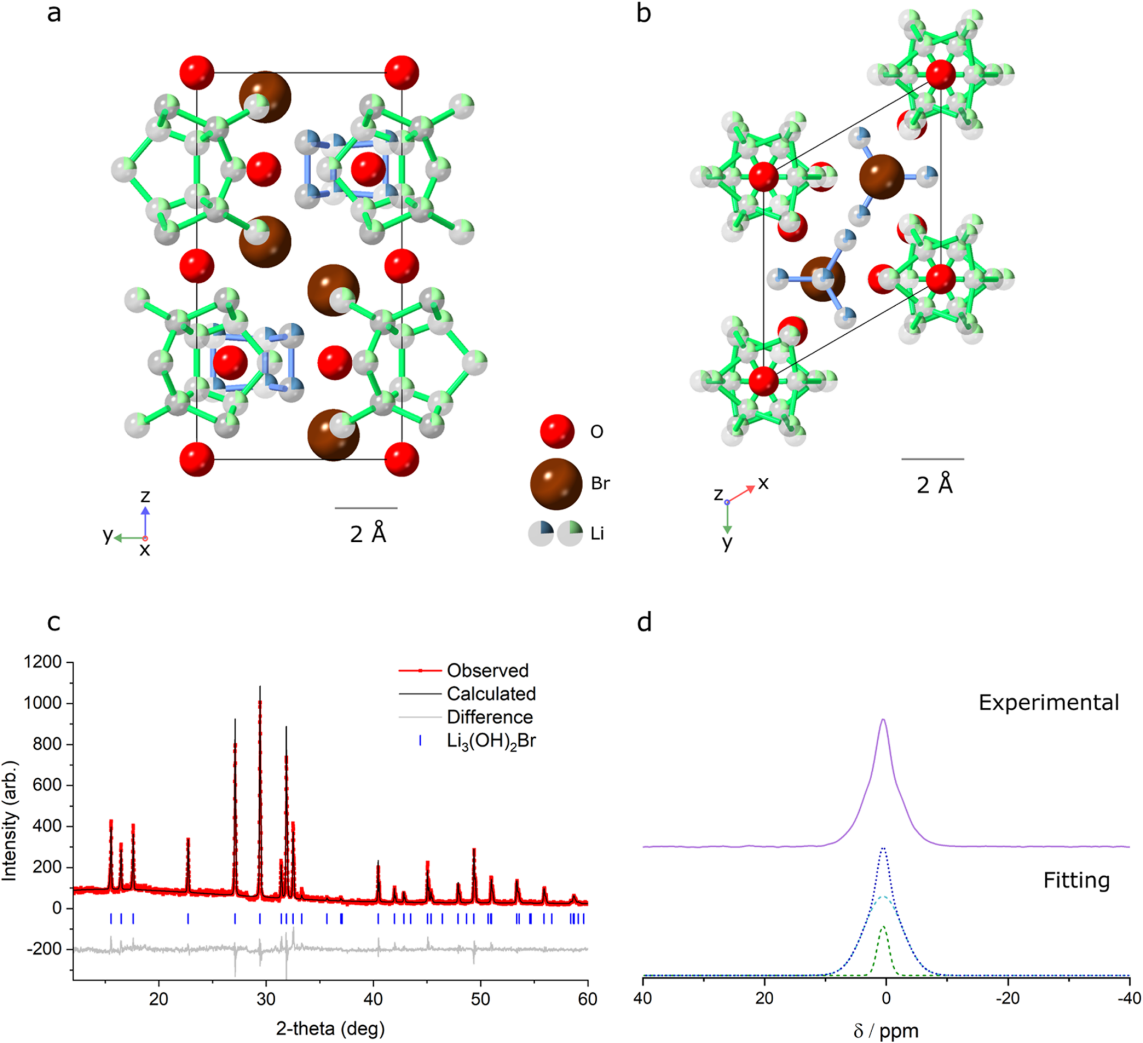
Crystal structure. Refined *P*6_3_/*mmc* structure for Li_3_(OH)_2_Br viewed along (a) [100] and (b) [001]. Lithium occupancies are represented by pie charts and are assumed to be equal (0.24) across all sites. Blue and green lithium atoms are used to distinguish between cage-like (green) and network-like (blue) sites in the structure. The green lithium atoms are extended beyond a single unit cell in the *a*–*b* plane to emphasize the cage-like structures they form. (c) XRD pattern of Li_3_(OH)_2_Br after 1 h annealing at 250 °C, with matching Rietveld refinement using the proposed *P*6_3_/*mmc* structural model of Li_3_(OH)_2_Br (*R*_wp_ = 11.9%). The corresponding difference curve is offset below the data, and ticks indicate the positions of the Bragg reflections. (d) ^7^Li NMR spectra of Li_3_(OH)_2_Br at 230 °C. The line shape can be deconvoluted into two Gaussian peaks, indicating 2 lithium environments in the structure.

The pseudosymmetry observed in the structure and the absence of a clear mechanism for inversion-symmetry breaking indicate that the phase may belong to a higher symmetry space group than *P*6_3_. The atomic positions were adjusted to be consistent with those of the *P*6_3_/*mmc* space group, which refined well with the observed diffraction data. The refined structural model for the proposed *P*6_3_/*mmc* structure is shown in Figure 2a,b, and compared with the computational starting model in Figure S4. A combination of network-like lithium sites and distinct “cage” geometries is predicted to exist in the structure, shown with blue and green lithium atoms, respectively. An XRD pattern taken at 250 °C, along with a corresponding Rietveld refinement, is shown in Figure 2c (Table 1). Information from the lithium in the structure was not refined due to its poor X-ray scattering cross section to avoid unphysical and inaccurate results. In order to establish information about lithium in the structure accurately, complementary techniques, such as neutron diffraction, will be necessary.

**Table 1:** Crystallographic Parameters from the Rietveld Refinement of Li_3_(OH)_2_Br In Situ at 250 °C Shown in Figure 2c^a^

space group	*P*6_3_/*mmc*
*a* = *b* (Å)	6.57137(8)
*c* (Å)	10.7454(2)
*V* (Å^3^)	401.851(13)

atom	Wyckoff position	*x*	*y*	*z*	occupancy	*U*_iso_ (Å^2^)
Br1	4f	1/3	2/3	0.9373(2)	1	0.0344(11)
O1	6h	0.1631(7)	0.8369	0.25	1	0.061(3)
O2	2a	0	0	0	1	0.061(3)
Li1	4e	0	0	0.3185	0.24	0.25(4)
Li2	6h	0.1775	0.8225	0.75	0.24	0.25(4)
Li3	12k	0.0938	0.9062	0.6454	0.24	0.25(4)
Li4	12k	0.1456	0.8544	0.0879	0.24	0.25(4)
Li5	4f	1/3	2/3	0.1905	0.24	0.25(4)
Li6	12k	0.0790	0.5395	0.1804	0.24	0.25(4)

a Errors on refined parameters are indicated in parentheses.

In addition to diffraction studies, ^7^Li NMR lineshapes were measured. Between 220 and 270 °C, in the temperature range of the Li_3_(OH)_2_Br phase, a line shape consisting of a narrow and a broad component is observed, which does not change significantly over the phase’s temperature range. An example spectrum taken at 230 °C is shown in Figure 2d. The narrow component suggests a highly mobile site, whereas the broad component indicates another less mobile environment. It is possible that the narrower element arises from the “cage” lithiums in the structure, in which rapid *intra*-cage hopping may occur.

### Lithium-Ion Dynamics

Another LiBr-LiOH compound, Li_2_OHBr, has high lithium-ion conductivity (∼10^–6^ S cm^–1^ at room temperature), making it of interest for solid-electrolyte applications.^1−5,18−20^ It is interesting to assess the mobility of lithium ions in Li_3_(OH)_2_Br to see whether it also exhibits superionic conductivity.

**Figure 3:**
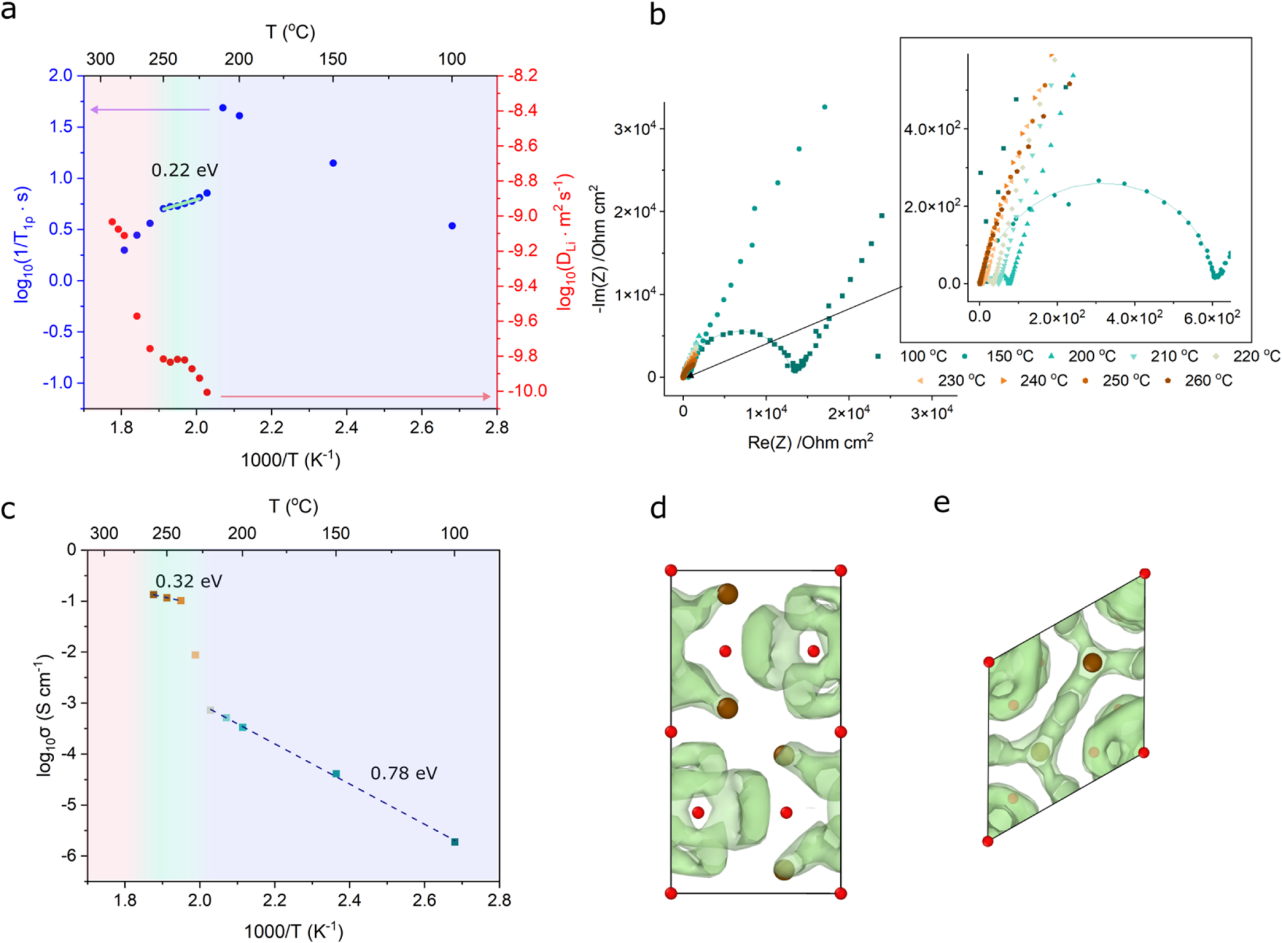
Li-ion dynamics. (a) NMR relaxometry and diffusivity measurements of 67 mol % LiOH stoichiometry sample as a function of temperature. Li_3_(OH)_2_Br forms from 220 °C and starts to melt from 260 °C. A linear fit to the SLR data is included for what is believed to be the pure Li_3_(OH)_2_Br phase region, used to calculate the activation energy for Li-ion hopping. (b) Nyquist plots from EIS of 67 mol % LiOH stoichiometry sample as a function of temperature. (c) Temperature dependence of ionic conductivity of 67 mol % LiOH stoichiometry sample. The ionic conductivity increases dramatically when Li_3_(OH)_2_Br forms above 225 °C. (d, e) Predicted lithium trajectories in the Li_3_(OH)_2_Br phase from molecular dynamics simulations, overlaid on the *P*6_3_/*mmc* unit cell and viewed along (d) [100] and (e) [001]. Lithium density is seen to be confined to cages with facile *intra*-cage hopping.

To evaluate the lithium-ion dynamics in the Li_3_(OH)_2_Br phase, we took NMR ^7^Li spin–lattice relaxation (SLR) measurements and pulsed-field gradient (PFG) NMR measurements across a range of temperatures, as shown in Figure 3a. The SLR measurements display three distinct regions. At low temperatures, the low-temperature flank of the rate peak corresponding to the regions of mixed Li_2_OHBr and Li_4_(OH)_3_Br can be seen. Around 220 °C, Li_3_(OH)_2_Br begins to form, and data points on the high-temperature flank of the corresponding rate peak can be seen. From 260 °C, another change corresponding to the sample melting is observed. The narrow temperature range in which the Li_3_(OH)_2_Br phase exists means that it is challenging to fit a Bloembergen–Purcell–Pound (BPP) model to the SLR data. To obtain an estimate of the activation energy for lithium-ion hopping, an approximation can instead be made using an Arrhenius relationship.^21,22^ A linear fit between 225 and 250 °C suggests a low activation energy of 0.22 eV. PFG-NMR measurements could be obtained from 220 °C (coinciding with the onset of Li_3_(OH)_2_Br formation). A lithium diffusivity of 1.53 × 10^–10^ m^2^ s^–1^ was found for the Li_3_(OH)_2_Br phase at 250 °C. Activation energy cannot be obtained from the diffusivity measurements due to microscopic changes occurring upon heating. The high temperatures involved result in sintering and grain growth in the powder sample, which impacts PFG diffusivity measurements probing a similar length scale.

To study ionic conductivity in Li_3_(OH)_2_Br over macroscopic length scales, EIS was conducted on cold-pressed pellets with stainless steel blocking electrodes and a high stack pressure of 70 MPa applied to minimize interfacial resistance. The Nyquist plots obtained are shown in Figure 3b. Characteristic semicircles are observed at lower temperatures, which could be fitted using an equivalent circuit containing a bulk and grain boundary component. As the temperature increases, low resistances result in the semicircles disappearing, and so the intersection of the low-frequency tail with the real axis was instead used to calculate the total resistance. The temperature dependence of the ionic conductivity is shown in Figure 3c. A step change corresponding to the phase transition forming Li_3_(OH)_2_Br can be seen at 230 °C, with an ionic conductivity of 0.12 S cm^–1^ at 250 °C and an activation energy of 0.32 ± 0.04 eV found between 240 and 260 °C. Upon extrapolating to room temperature, an ionic conductivity of 5.2 ± 1.6 × 10^–4^ S cm^–1^ is found. It would be desirable to retain this phase and hence the excellent ionic conductivity to lower temperatures. Li_3_(OH)_2_Br could offer several significant advantages over many solid electrolytes currently under consideration. Li_3_(OH)_2_Br contains inexpensive and abundant precursors, unlike popular options such as Li_7_La_3_Zr_2_O_12_ (LLZO) and lithium argyrodite sulfides.^23^ Li_2_OHBr exhibits good stability with lithium metal at room temperature, as is the case with other oxide electrolytes, which is anticipated to translate to Li_3_(OH)_2_Br.^3,5^ Furthermore, the density of Li_3_(OH)_2_Br (2.23 g cm^–3^) is significantly reduced compared to other oxide electrolytes, such as LLZO (5.07 g cm^–3^), Li_0.34_La_0.56_TiO_3_ (5.01 g cm^–3^), and Li_1.5_Al_0.5_Ge_1.5_(PO_4_)_3_ (3.56 g cm^–3^).^24^

To identify and eliminate the impacts of sintering occurring at high temperatures, EIS measurements were also taken using another heating protocol (see Supporting Note 1). Similar behavior was observed for the Li_3_(OH)_2_Br phase in both samples (Figure S5), indicating that the obtained conductivity is representative of that of bulk Li_3_(OH)_2_Br.

Molecular dynamics simulations were carried out on a supercell of the computational Li_3_(OH)_2_Br structure containing 576 Li ions across a temperature range of 220 to 240 °C. Trajectories, shown in Figure 3d,e, reveal that the Li density is largely confined to “cages” in which Li can easily move between sites within the cage (*intra*-cage), but where jumps between the cages (*inter*-cage) are less frequent. The corresponding activation energies for intracage and intercage jumps were calculated to be 0.18 ± 0.02 and 0.35 ± 0.02 eV respectively. Accordingly, the macroscopic activation energy is calculated to be 0.27 ± 0.03 eV, which is in reasonable agreement with the EIS findings. The underestimate in activation energy between MD and EIS is reasonable given the differences between the computational and experimentally refined structure and the absence of extended defects such as grain boundaries in MD, which assumes a pristine crystal. Attempts to engineer Li_3_(OH)_2_Br to improve ionic conductivity may focus on lowering the barriers for intercage diffusion to enable long-range transport. Discrepancies between the intra- and intercage activation energies are well studied in the familiar argyrodite family of solid electrolytes Li_6_PS_5_Cl,^25^ where increased disorder is a possible avenue to encouraging macroscopic diffusion.

### Metastable Retention of Li_3_(OH)_2_Br

To see whether the Li_3_(OH)_2_Br phase could be retained at room temperature metastably, samples were annealed at 250 °C for 4 h such that Li_3_(OH)_2_Br fully forms, followed by quenching to room temperature to try and “freeze-in” the phase. Both samples quenched from the liquid state at 400 °C and from the Li_3_(OH)_2_Br phase field at 250 °C exhibited a similar XRD pattern with a different set of reflections to Li_3_(OH)_2_Br, Li_2_(OH)Br, or Li_4_(OH)_3_Br (Figure S6a). The thermodynamically unstable nature of the *P*6_3_/*mmc* phase at low temperatures, coupled with the fast cooling rate inhibiting the necessary atomic rearrangements for attaining equilibrium, results in the metastable state observed here. The ionic conductivity of this metastable state was assessed using EIS (Figure S6b,c), but did not demonstrate the same promising behavior as the high-temperature phase, yielding an ionic conductivity of 3.6 × 10^–8^ S cm^–1^ at 30 °C and an activation energy of 0.75 eV in the temperature range 30 to 50 °C. DC chronoamperometry measurements indicated an electronic conductivity of 2.0 × 10^–11^ S cm^–1^ at 25 °C (Figure S6d). This ionic conductivity is lower than that of the related compound and solid-electrolyte candidate, Li_2_OHBr, which typically exhibits ionic conductivities of ∼10^–6^ S cm^–1^ for cold-pressed pellets at room temperature.^1−5,18−20^ Synthesis of Li_2_OHBr from the melt, as is typical in the literature, requires cooling through phase fields containing Li_3_(OH)_2_Br (Figure 1a). Consequently, it is important to consider undesirable Li_3_(OH)_2_Br impurity formation in the synthesis of Li_2_OHBr. The small crystallite sizes formed during cooling, combined with potentially high levels of microstrain from the diffusionless metastable transformation, may mean that it is not obvious in XRD, typically used to screen for impurities.

## Conclusions

In this work, we investigated the high-temperature Li_3_(OH)_2_Br phase which is thermodynamically stable between ∼225 and 275 °C. Diffraction studies suggest the phase takes a hexagonal unit cell with lattice parameters of *a* = *b* = 6.572 Å and *c* = 10.746 Å at 250 °C. Through a combination of XRD refinements and theoretical prediction, a structural model with the *P*6_3_/*mmc* space group is suggested. MD indicates that the structure contains cage-like structures of lithium, in which intracage diffusion is facile. A macroscopic ionic conductivity of 0.12 S cm^–1^ is measured at 250 °C, which would extrapolate to 5 × 10^–4^ S cm^–1^ upon retention at room temperature. However, attempts to stabilize this phase were not successful, resulting in a different metastable state with a worse ionic conductivity.

## Experimental Methods

### Synthesis

For the synthesis described in the main text, anhydrous LiOH (98%, Sigma-Aldrich) and LiBr (≥99%, Sigma-Aldrich) were used. Synthesis was carried out in an MTI compact muffle furnace inside an argon-filled glovebox (MBraun, H_2_O < 0.5 ppm, O_2_ < 0.5 ppm). All utensils and consumables were dried in a vacuum oven (∼1 mbar, 70 °C) for at least 4 h prior to use.

### XRD

Room-temperature XRD measurements were taken using a Rigaku Miniflex diffractometer (Cu Kα) inside a nitrogen-filled glovebox (MBraun, H_2_O < 0.5 ppm, O_2_ < 0.5 ppm). Powder samples were loaded onto a single-crystal silicon holder to minimize background contributions. VT XRD measurements in Figure 1d and Figure S2a were carried out on the I11 beamline at Diamond Light Source (λ = 0.82311 Å) on powders sealed in borosilicate glass capillaries under argon and heated continuously at 6 °C/min using an FMB Oxford cyberstar hot air blower. Diffraction patterns were measured in capillary transmission geometry using a Mythen2 Position Sensitive Detector. Two data collections, each 5 s, were taken at angles 0.25° apart and summed to account for gaps in detector coverage. The in situ 250 °C measurements used for refinements were taken on a Rigaku Smartlab diffractometer (Cu Kα), following a heating step at 1 °C/min and a holding step for 1 h at 250 °C. For this, the powder was pressed into a 10 mm diameter pellet and loaded onto a heating stage under argon flow.

Pawley and Rietveld refinements were conducted using TOPAS-Academic software.^16^ In both, the unit cell, background, and peak shape parameters were allowed to refine freely. In Rietveld refinements, the O and Br fractional positions were allowed to refine, in addition to 3 thermal displacement parameters for the Br, O, and Li atoms.

### NMR

^7^Li NMR measurements were performed using an ECZ-500 (JEOL, Japan) spectrometer and a homemade high-temperature PFG-NMR probe.^26^ The sample was packed into a quartz NMR tube SP-405 (SHIGEMI, Japan) and sealed in an Ar-filled glovebox. The resonance frequency of ^7^Li was 194.4 MHz. NMR spectra were recorded by using a single-pulse sequence. The chemical shift was referenced to a 1.0 M LiCl solution at 0 ppm. Spin–lattice relaxation times were measured by using a saturation recovery method. The data was fitted to *f*(*t*) = *f*_∞_(1 – *H*_0_ exp(−*t/T*_1ρ_)). The diffusion coefficients were measured by using the STE-PFG sequence.

### DSC

DSC measurements were taken under argon on a Netzsch TGA-MS using ∼5 mg of sample in an alumina pan covered with an alumina lid. An argon shower was used to protect the sample from air exposure during loading into the instrument. A heating and cooling rate of 5 °C/min was used. Background subtraction was carried out using OriginLab software.

### Electrochemical Measurements

For electrochemical measurements, the powder samples were pressed into 5 mm diameter pellets for 3 min at 370 MPa. For measurements of the quenched metastable state, nickel foil (Advent Materials, 99.95%, 0.0125 mm) blocking electrodes were placed on either side of the electrolyte pellet. For high-temperature measurements of the Li_3_(OH)_2_Br phase, stainless steel pistons were used as blocking electrodes due to the reactivity of nickel. A custom-built cell applied a uniaxial pressure of 70 MPa to ensure good contact between the electrolyte and blocking electrodes. Assembly and measurements were carried out in an argon-filled glovebox.

For EIS measurements, the cells were heated in an MTI compact muffle furnace. The samples were held at the desired temperature for 30 min prior to measurement, followed by a 15 min ramp period to the next temperature. Measurements were taken using a BioLogic MTZ35 frequency response analyzer in a two-point probe configuration in the frequency range 35 MHz to 0.1 Hz with a voltage amplitude of 10 mV. Conductivities were obtained from Nyquist plots. For measurements of the quenched metastable state, equivalent circuits consisting of a resistor and contributions corresponding to bulk and grain boundary resistances, *R*_1_ + *Q*_2_/*R*_2_ + *Q*_3_/*R*_3_, were used to model the data using BioLogic EC lab software. DC chronoamperometry measurements were taken for 10 h at 0.3, 0.6, and 1 V.

## Computational Methods

A preliminary ab initio random structure searching (AIRSS)^27^ run was performed on several hundred geometries for Li_3_(OH)_2_Br and Li_4_(OH)_3_Br using the pretrained CHGNet foundation model. Then, a CHGNet model was fine-tuned for Li–O–H–Br systems by training them against a set of molecular dynamics trajectories. The systems were: solid Li_2_OHBr, Li_3_(OH)_2_Br, and Li_4_(OH)_3_Br at 900 K with *NPT* ensemble; and molten Li_2_OHBr, Li_3_(OH)_2_Br, and Li_4_(OH)_3_Br at 2000 K with *NVT* ensemble. The fine-tuned model was then used for the final AIRSS to determine the stable structure in this work and for all further MD runs. Due to the importance of H-bonding in materials containing OH species, dispersion was treated using the DFT-D3 method^28^ using the implementation in torch-dftd.^29^ Analysis of the MD trajectories was performed with Pymatgen(30) and Gemdat,^31^ which allows for decomposition of activation energies into distinct hops between atomic sites.

Fully occupied unit cells were produced by structure searching. Then, a supercell was constructed and equilibrated at 250 °C. Partially occupied sites were determined by wrapping the trajectory back into the unit cell to determine the stable sites. Because Li diffuses so readily in the structure, the convergence of the site occupancies is poor. For simplicity, the occupation of the determined stable sites was normalized to give the correct number of Li atoms per unit cell.

All geometry optimizations were carried out until the forces on the ions were less than 0.05 eV/Å. The time step for molecular dynamics was 0.5 fs to ensure numerical stability when equations of motion were integrated due to the small mass and rapid acceleration of protons. The computational activation energy was fitted to temperatures of 220, 230, 230, 235, 240, 245, 250, 260, and 270 °C. All trajectories are at least 250 ps long. For fitting to the Einstein relation, the trajectories were split into 5 parts to calculate mean-squared displacements in order to reduce errors arising from deviations from linearity in long trajectories.^32^ Simulated ab initio MD for fine-tuning was performed in VASP. Exchange correlation was treated with the PBE functional.^33^ Projector-augmented wave (PAW) pseudopotentials^34,35^ were employed in which the following electrons were treated as valence: 1s^1^ for H, 1s^2^2s^1^ for Li; 2s^2^2p^4^ for O; and 4s^2^4p^5^ for Br. The plane-wave basis used a cutoff energy of 500 eV.

## Supporting Information Available

Additional computational and experimental results including XRD, Raman spectroscopy, NMR, and EIS (PDF)
