# Supplementary material for: Filling the Gaps in the LiBr-LiOH Phase Diagram: A Study on the High-Temperature Li3(OH)2Br Phase
Source: Chem Mater. 2025 Apr 4;37(8):2899–906. doi: 10.1021/acs.chemmater.5c00206 (PMC12019999; doi:10.1021/acs.chemmater.5c00206)
Supplement: Supplementary file 1 — cm5c00206_si_001.pdf [file cm5c00206_si_001.pdf]

Filling the gaps in the LiBr-LiOH phase diagram: A study on the high-temperature $Li_3(OH)_2Br$ phase - SUPPORTING INFORMATION

Emily Milan[1], James A. Quirk[2], Kenjiro Hashi[3], John Cattermull[1,4], Andrew L. Goodwin[4], James A. Dawson[2], and Mauro Pasta*[1]

[1]Department of Materials, University of Oxford, Oxford, OX1 3PH, UK

[2]Chemistry – School of Natural and Environmental Sciences, Newcastle University, Newcastle upon Tyne, NE1 7RU, UK

[4]Department of Chemistry, University of Oxford, Oxford, OX1 3QR, United Kingdom

[3]National Institute for Materials Science, Tsukuba, 305-0044, Japan

*Corresponding author: mauro.pasta@materials.ox.ac.uk

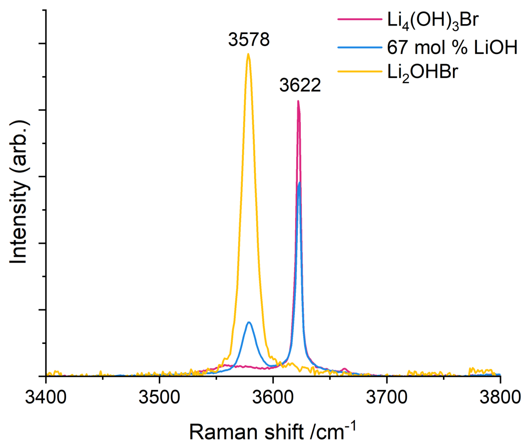

Figure S1: Room-temperature Raman spectroscopy measurements in the O-H bond stretching vibration region, showing 67 mol % LiOH sample cooled from 350 °C at 3 °C/min contains bonding environments corresponding to Li_2OHBr (3578 cm^{-1}) and $Li_4(OH)_3Br$ (3622 cm^{-1}), as expected from Mahroug's phase diagram.

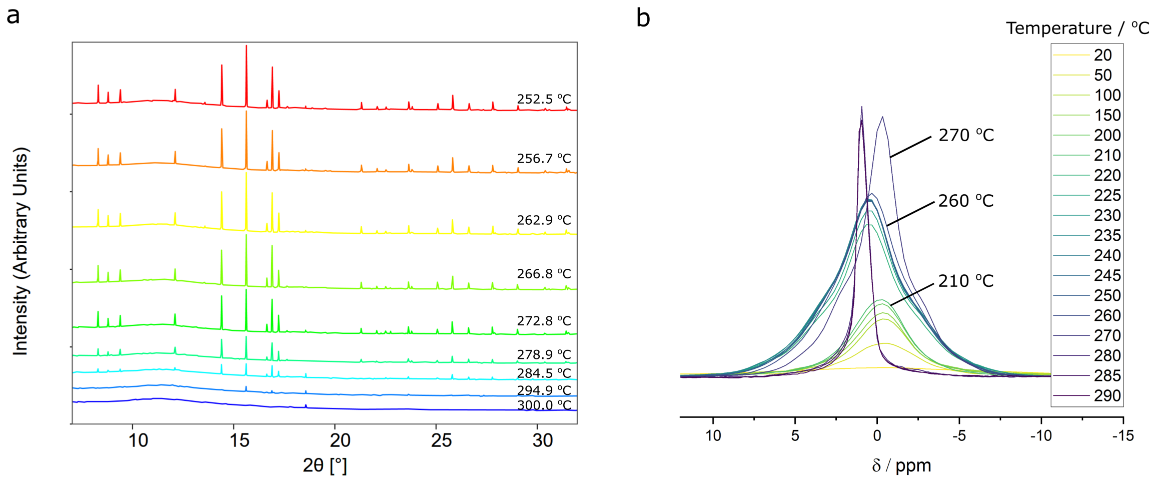

Figure S2: $Li_3(OH)_2Br$ Melting. a) In-situ synchrotron XRD heating of $Li_3(OH)_2Br$ at 6 °C/min. The decrease in peak intensity corresponds to sample melting. The bottom spectrum corresponds to the sample after holding at 300 °C for 10 minutes. Residual solid at 300 °C is expected to be LiOH from the existing phase diagram, but these peaks correspond to another, unidentified phase. b) ^{7}Li NMR lineshapes taken at temperatures between 20 °C and 290 °C, showing a phase transition at ∼ 220 °C and ∼ 260 °C

Table S1: XRD peak positions (CuKα) corresponding to the $Li_3(OH)_2Br$ phase at 250 °C, used to search for the lattice parameters and space group of the $Li_3(OH)_2Br$ phase.

Peak Position / $2\theta(^o)$
15.55
16.49
17.62
22.74
27.11
29.44
31.41
31.89
32.52
33.32

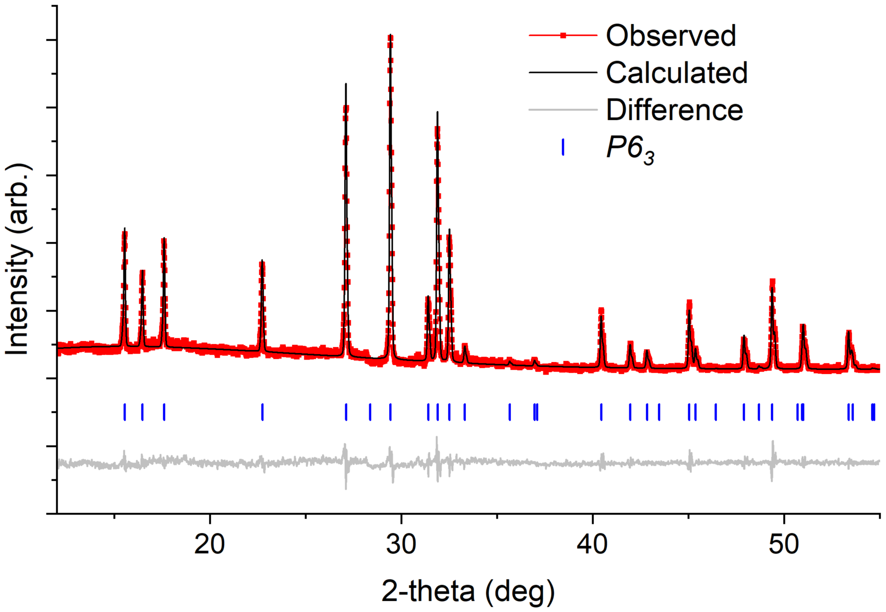

Figure S3: XRD pattern of $Li_3(OH)_2Br$ at 250 °C and corresponding Pawley refinement (R_{wp} = 10.5%) for the $P6_3$ space group. The corresponding difference curve is offset below the data. Bragg peak positions expected from the space group symmetry are shown with ticks.

Table S2: Unit cell parameters determined from the Pawley fit shown in Figure S3, for the $P6_3$ structure of $Li_3(OH)_2Br$ at 250 °C.

Unit Cell	
$a = b$ (Å)	6.57192(6)
c (Å)	10.74643(17)
$\alpha = \beta$ (°)	90
γ (°)	120
V (Å^3)	401.959(10)

Table S3: Crystallographic parameters from the computationally-determined $P6_3$ $Li_3(OH)_2Br$ crystal structure, shown in Figure S4.

Space Group	$P6_3$				
$a = b$ (Å)	6.437163				
c (Å)	10.617548				
V (Å^3)	381.017				
Atom	Wyckoff position	x	y	z	Occupancy
Br1	2b	1/3	2/3	0.913445	1
Br2	2b	1/3	2/3	0.282528	1
O1	6c	0.147307	0.804925	0.592446	1
O2	2a	1	0	0.814739	1
Li1	6c	0.529535	0.470658	0.0354630	0.214
Li2	6c	0.5496650	0.449422	0.174301	0.214
Li3	2b	2/3	1/3	0.027143	0.214
Li4	2b	2/3	1/3	0.146215	0.214
Li5	6c	0.770769	0.707472	0.917378	0.214
Li6	6c	0.941172	0.709313	0.919614	0.214
Li7	6c	0.111691	0.899328	0.984623	0.214
Li8	6c	0.180061	0.354849	0.091703	0.214
Li9	6c	0.872638	0.709476	0.258420	0.214
Li10	6c	0.818558	0.905321	0.193865	0.214
Li11	2a	0	0	0.525085	0.214
Li12	2a	0	0	0.661937	0.214

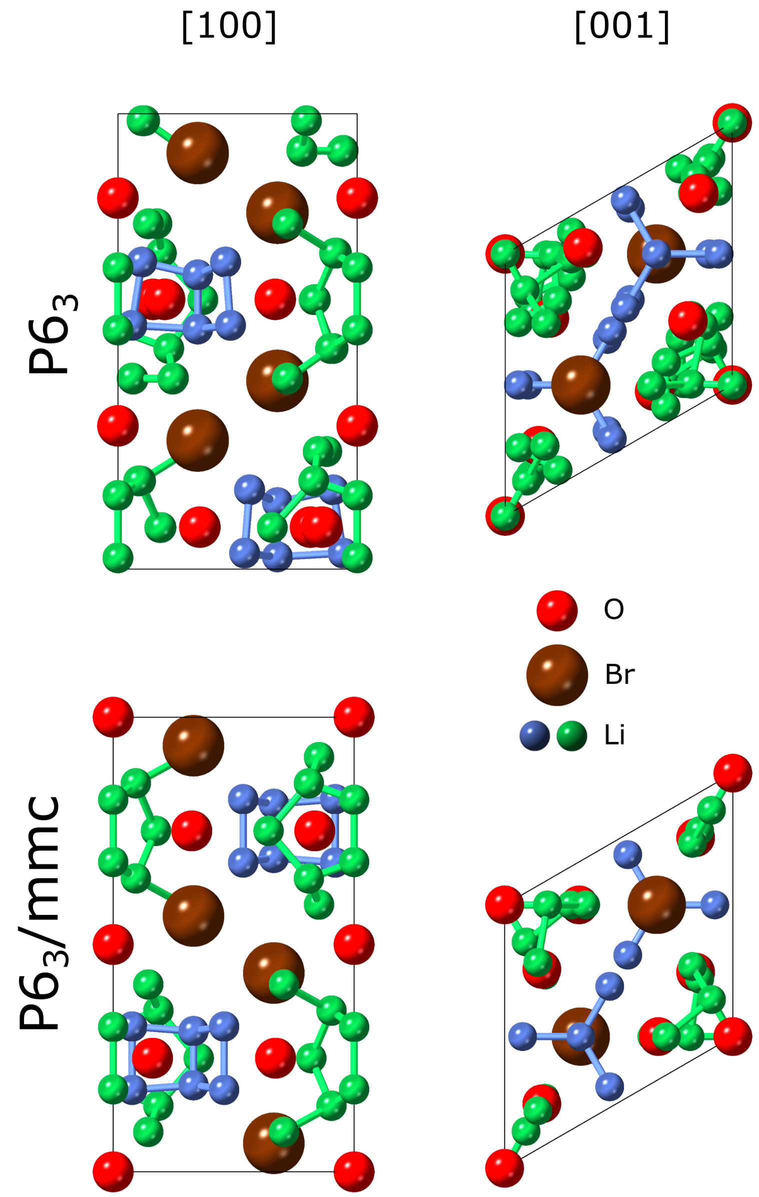

Figure S4: Comparison of the computationally-determined $P6_3$ model for $Li_3(OH)_2Br$ (top) and the final refined $P6_3/mmc$ structure (bottom), viewed along [100] and [001]. Note that the origin of the $P6_3/mmc$ unit cell is shifted along the c-axis relative to the $P6_3$ structure to satisfy the additional symmetry constraints. Lithium sites are depicted as fully occupied for easier visualization, but are expected to correspond to 0.214 in the $P6_3$ structure and 0.24 in the $P6_3/mmc$ structure.

Supplementary Note 1

Identical samples of 67 mol % LiOH stoichiometry, cooled to room temperature from 350 °C at 3 °C/min and ground into a powder, were measured by EIS. In both cases, samples were held at the desired temperature for 30 minutes prior to measurement, followed by a 15 minute ramp period to the next temperature. Sample 1 (shown in the main text) was measured at the following temperatures consecutively: 25 °C, 100 °C, 150 °C, 200 °C, 210 °C, 220 °C, 230 °C, 240 °C, 250 °C, 260 °C. Sample 2 underwent additional cooling stages at 250 °C, 240 °C, 230 °C, 220 °C and 210 °C following the heating measurements. Arrhenius plots of the total conductivities obtained from these measurements are shown in Figure S5. Note that the heating measurement at 240 °C on sample 2 failed, and so does not appear in the plot.

A slight increase in ionic conductivity can be seen between the heating and cooling cycles. This is likely due to the additional sintering and grain growth occurring during the extended periods at elevated temperatures.

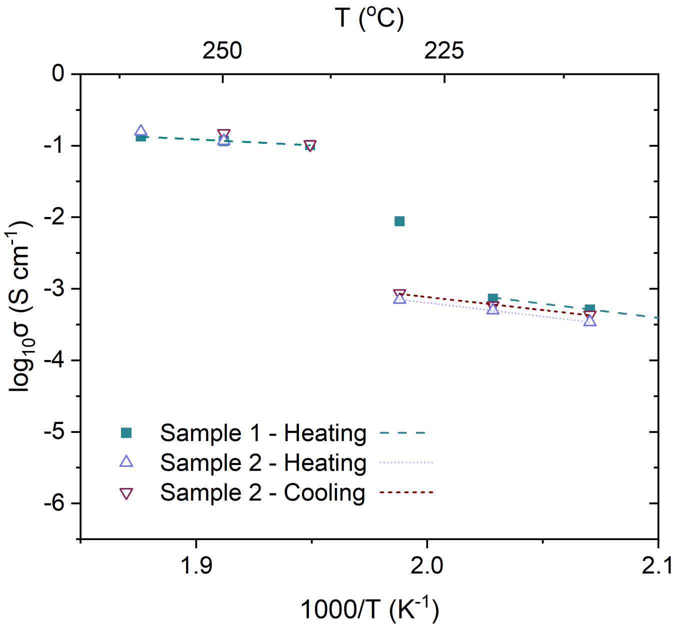

Figure S5: Arrhenius plot showing the ionic conductivity of cold-pressed $Li_3(OH)_2Br$ samples upon heating and cooling, to establish whether microstructural changes occurring upon heating impact the measurements. The conductivity measurements in the $Li_3(OH)_2Br$ phase field are very similar upon heating and cooling, indicating that microstructural elements such as grain boundary resistance are not dominating the observed behavior.

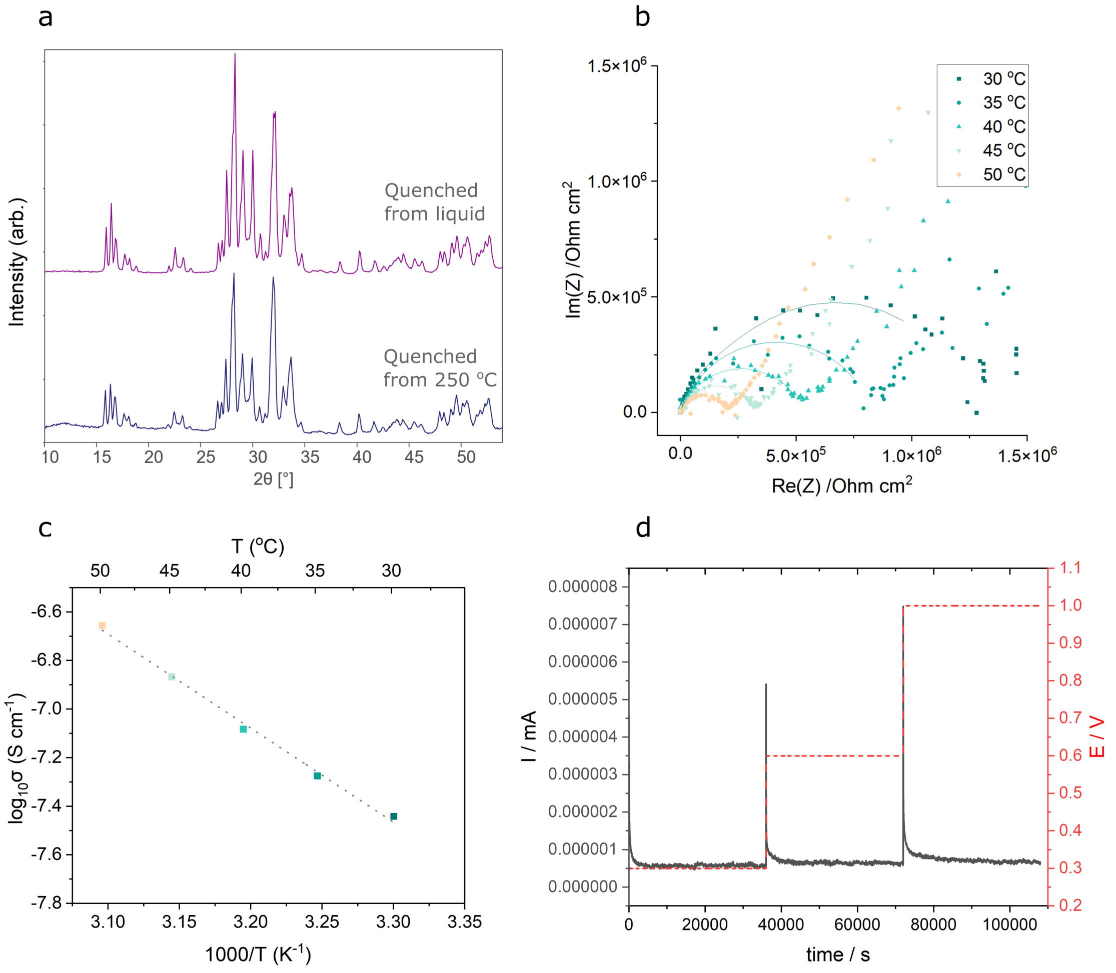

Figure S6: Quenched $Li_3(OH)_2Br$. a) XRD spectra of 67 mol % LiOH after quenching to room temperature from the liquid state (at 400 °C), and from an anneal at 250 °C. The resulting patterns correspond to a metastable state not containing the $Li_3(OH)_2Br$ phase observed above ∼ 230 °C, $Li_4(OH)_3Br$ or Li_2OHBr. b) Nyquist plots from electrochemical impedance spectroscopy of quenched $Li_3(OH)_2Br$ with Ni-foil blocking electrodes as a function of temperature. b) Temperature dependence of the ionic conductivity. c) Room temperature DC chronoamperometry used for determination of the electronic conductivity
